# *N*-Morpholino-Δ^8^-dihydro­abietamide

**DOI:** 10.1107/S1600536810039073

**Published:** 2010-10-09

**Authors:** Xiao-Ping Rao

**Affiliations:** a Institute of Chemical Industry of Forest Products, Chinese Academy of Forestry, Nanjing 210042, People’s Republic of China

## Abstract

The title compound, C_24_H_39_NO_2_ (systematic name: 4-{[1,4a-dimethyl-7-(propan-2-yl)-1,2,3,4,4a,5,6,7,8,9,10,10a-dodeca­hydro­phenanthren-1-yl]carbon­yl}morpholine), has been synthesized from Δ^8^-dihydro­abietic acid. Two cyclo­hexene rings adopt half-chair conformations, whereas the cyclo­hexane and morpholine rings are each in the chair conformation. Two methyl groups are in an axial position with respect to the tricyclic hydro­phenanthrene nuclei.

## Related literature

For literature on Δ^8^-dihydro­abietic acid, see: Rao *et al.* (2009 ▶). For the biological activity of rosin acid derivatives, see Fonseca *et al.* (2004 ▶); Sepulveda *et al.* (2005 ▶).
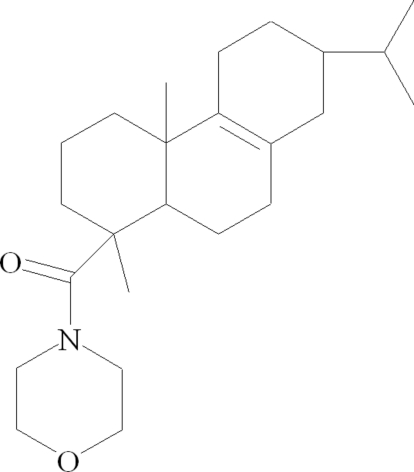


## Experimental

### 

#### Crystal data

C_24_H_39_NO_2_
*M* *_r_* = 373.56
Orthorhombic, 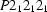

*a* = 7.8683 (16) Å
*b* = 11.036 (2) Å
*c* = 24.726 (5) Å
*V* = 2147.1 (7) Å^3^
*Z* = 4
Mo *K*α radiation
μ = 0.07 mm^−1^
*T* = 293 K
0.42 × 0.34 × 0.25 mm

#### Data collection

Rigaku R-AXIS RAPID diffractometer
Absorption correction: multi-scan (*ABSCOR*; Higashi, 1995 ▶) *T*_min_ = 0.352, *T*_max_ = 0.497
16937 measured reflections
2186 independent reflections
1957 reflections with *I* > 2σ(*I*)
*R*_int_ = 0.029

#### Refinement

*R*[*F* ^2^ > 2σ(*F* ^2^)] = 0.038
*wR*(*F* ^2^) = 0.117
*S* = 1.37
2186 reflections
248 parameters
H-atom parameters constrained
Δρ_max_ = 0.23 e Å^−3^
Δρ_min_ = −0.26 e Å^−3^

### 

Data collection: *RAPID-AUTO* (Rigaku, 1998 ▶); cell refinement: *RAPID-AUTO*; data reduction: *RAPID-AUTO*; program(s) used to solve structure: *SHELXS97* (Sheldrick, 2008 ▶); program(s) used to refine structure: *SHELXL97* (Sheldrick, 2008 ▶); molecular graphics: *XP* (Sheldrick, 2008 ▶); software used to prepare material for publication: *SHELXL97*.

## Supplementary Material

Crystal structure: contains datablocks I, global. DOI: 10.1107/S1600536810039073/kp2277sup1.cif

Structure factors: contains datablocks I. DOI: 10.1107/S1600536810039073/kp2277Isup2.hkl

Additional supplementary materials:  crystallographic information; 3D view; checkCIF report

## Figures and Tables

**Fig. 1.**
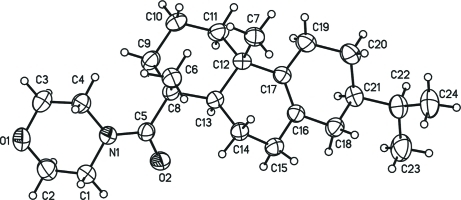
Molecular structure of the title compound, with H atoms represented by small spheres of arbitrary radius and displacement ellipsoids at the 30% probability level.

## supplementary crystallographic information

### Comment

Δ^8^-Dihydroabietic acid is one of the main component of hydrogenated rosin. It is more stable to air oxidation than abietic acid (Rao *et al.*, 2009). Rosin acid derivatives exhibit wide range of biological activities, such as antifungal and antitumor (Fonseca *et al.*, 2004), nitrogen derivatives of rosin acid have been studied as gastroprotective and cytotoxic reagents and they are found to have high activity in reducing blood serum cholesterol levels in animals (Sepulveda *et al.*, 2005). In this work, we describe the crystal structure of the title compound.

Two cyclohexene rings adopt a half-chair conformations, and the cyclohexane and morpholine rings are in the chair conformation. Two methyl groups are in an axial position of the tricyclic hydrophenanthrene nuclei. The absolute configuration cannot be assigned on a basis of the value of the Flack parameter due to its high deviation. The Friedel opposite reflections were not measured.

### Experimental

A mixture of Δ^8^-dihydroabietic acid (0.1 mol), trichloro phosphorous (6 ml) and chloroform (40 ml) was stirred at 333 K for 3 h, after distilled off the solvent, the residue was added to the morpholine (0.2 mol) in toluene (60 ml) solution, the mixture was reacted for 24 h at room temperature, then the solvent was distilled off, upon recrystallization from acetone, white crystals of the title compound were obtained (yield 40%, m.p.394 K). Single crystals were grown from acetone.

### Refinement

H atoms were positioned geometrically and refined as riding atoms, with C—H = 0.96Å and *U*_iso_(H) = 1.5*U*_eq_(C) for methyl H atoms, and C—H = 0.97–0.98Å and *U*_iso_(H) = 1.2*U*_eq_(C) for all other H atoms.

### Crystal data

**Table:**

C_24_H_39_NO_2_	*F*(000) = 824
*M**_r_* = 373.56	*D*_x_ = 1.156 Mg m^−^^3^
Orthorhombic, *P*2_1_2_1_2_1_	Mo *K*α radiation, λ = 0.71073 Å
Hall symbol: P 2ac 2ab	Cell parameters from 16603 reflections
*a* = 7.8683 (16) Å	θ = 3.1–27.4°
*b* = 11.036 (2) Å	µ = 0.07 mm^−^^1^
*c* = 24.726 (5) Å	*T* = 293 K
*V* = 2147.1 (7) Å^3^	Block, colorless
*Z* = 4	0.42 × 0.34 × 0.25 mm

### Data collection

**Table:**

Rigaku R-AXIS RAPID diffractometer	2186 independent reflections
Radiation source: fine-focus sealed tube	1957 reflections with *I* > 2σ(*I*)
graphite	*R*_int_ = 0.029
Detector resolution: 0 pixels mm^-1^	θ_max_ = 25.0°, θ_min_ = 3.1°
ω scans	*h* = −9→9
Absorption correction: multi-scan (*ABSCOR*; Higashi, 1995)	*k* = −13→13
*T*_min_ = 0.352, *T*_max_ = 0.497	*l* = −29→29
16937 measured reflections	

### Refinement

**Table:**

Refinement on *F*^2^	Secondary atom site location: difference Fourier map
Least-squares matrix: full	Hydrogen site location: inferred from neighbouring sites
*R*[*F*^2^ > 2σ(*F*^2^)] = 0.038	H-atom parameters constrained
*wR*(*F*^2^) = 0.117	*w* = 1/[σ^2^(*F*_o_^2^) + (0.0672*P*)^2^] where *P* = (*F*_o_^2^ + 2*F*_c_^2^)/3
*S* = 1.37	(Δ/σ)_max_ < 0.001
2186 reflections	Δρ_max_ = 0.23 e Å^−^^3^
248 parameters	Δρ_min_ = −0.26 e Å^−^^3^
0 restraints	Absolute structure: Flack (1983), ??? Friedel pairs
Primary atom site location: structure-invariant direct methods	Flack parameter: 0 (10)

### Special details

**Table:**

Geometry. All e.s.d.'s (except the e.s.d. in the dihedral angle between two l.s. planes) are estimated using the full covariance matrix. The cell e.s.d.'s are taken into account individually in the estimation of e.s.d.'s in distances, angles and torsion angles; correlations between e.s.d.'s in cell parameters are only used when they are defined by crystal symmetry. An approximate (isotropic) treatment of cell e.s.d.'s is used for estimating e.s.d.'s involving l.s. planes.
Refinement. Refinement of *F*^2^ against ALL reflections. The weighted *R*-factor *wR* and goodness of fit *S* are based on *F*^2^, conventional *R*-factors *R* are based on *F*, with *F* set to zero for negative *F*^2^. The threshold expression of *F*^2^ > σ(*F*^2^) is used only for calculating *R*-factors(gt) *etc*. and is not relevant to the choice of reflections for refinement. *R*-factors based on *F*^2^ are statistically about twice as large as those based on *F*, and *R*- factors based on ALL data will be even larger.

### Fractional atomic coordinates and isotropic or equivalent isotropic displacement parameters (Å^2^)

**Table:**

	*x*	*y*	*z*	*U*_iso_*/*U*_eq_	
O1	1.1743 (3)	0.85945 (18)	0.01582 (7)	0.0661 (6)	
O2	0.9634 (2)	0.83691 (14)	0.19288 (7)	0.0528 (5)	
N1	1.0754 (3)	0.75942 (19)	0.11701 (8)	0.0523 (6)	
C1	1.1939 (4)	0.8606 (3)	0.11335 (10)	0.0577 (7)	
H1A	1.3095	0.8301	0.1132	0.069*	
H1B	1.1805	0.9126	0.1447	0.069*	
C2	1.1629 (4)	0.9328 (3)	0.06276 (10)	0.0627 (8)	
H2A	1.0508	0.9693	0.0645	0.075*	
H2B	1.2458	0.9977	0.0605	0.075*	
C3	1.0545 (4)	0.7641 (3)	0.01898 (10)	0.0586 (7)	
H3A	1.0628	0.7147	−0.0134	0.070*	
H3B	0.9409	0.7981	0.0203	0.070*	
C4	1.0817 (4)	0.6852 (2)	0.06789 (9)	0.0501 (6)	
H4A	0.9944	0.6233	0.0694	0.060*	
H4B	1.1913	0.6453	0.0654	0.060*	
C5	0.9656 (3)	0.75310 (19)	0.16034 (9)	0.0404 (5)	
C6	0.9513 (3)	0.52216 (19)	0.16245 (10)	0.0467 (6)	
H6A	1.0593	0.5390	0.1460	0.070*	
H6B	0.8898	0.4655	0.1404	0.070*	
H6C	0.9688	0.4882	0.1978	0.070*	
C7	0.6449 (4)	0.4303 (2)	0.23662 (10)	0.0492 (6)	
H7A	0.7448	0.4175	0.2582	0.074*	
H7B	0.6643	0.4004	0.2007	0.074*	
H7C	0.5508	0.3879	0.2525	0.074*	
C8	0.8477 (3)	0.64162 (18)	0.16745 (8)	0.0371 (5)	
C9	0.7048 (3)	0.6567 (2)	0.12412 (9)	0.0467 (6)	
H9A	0.6655	0.7400	0.1246	0.056*	
H9B	0.7526	0.6410	0.0886	0.056*	
C10	0.5535 (3)	0.5732 (2)	0.13276 (10)	0.0509 (6)	
H10A	0.4685	0.5895	0.1053	0.061*	
H10B	0.5899	0.4898	0.1287	0.061*	
C11	0.4745 (3)	0.5901 (2)	0.18848 (9)	0.0478 (6)	
H11A	0.3801	0.5343	0.1926	0.057*	
H11B	0.4302	0.6718	0.1915	0.057*	
C12	0.6045 (3)	0.56781 (19)	0.23426 (9)	0.0373 (5)	
C13	0.7604 (3)	0.65211 (18)	0.22398 (8)	0.0344 (5)	
H13A	0.7140	0.7345	0.2250	0.041*	
C14	0.8810 (3)	0.6454 (2)	0.27262 (9)	0.0391 (5)	
H14A	0.9105	0.5616	0.2799	0.047*	
H14B	0.9847	0.6895	0.2648	0.047*	
C15	0.7940 (3)	0.7000 (2)	0.32151 (9)	0.0448 (6)	
H15A	0.7983	0.7876	0.3187	0.054*	
H15B	0.8562	0.6768	0.3538	0.054*	
C16	0.6101 (3)	0.66108 (19)	0.32775 (9)	0.0376 (5)	
C17	0.5253 (3)	0.60145 (18)	0.28917 (9)	0.0381 (5)	
C18	0.5310 (3)	0.6973 (2)	0.38098 (9)	0.0456 (6)	
H18A	0.5529	0.7828	0.3869	0.055*	
H18B	0.5875	0.6530	0.4097	0.055*	
C19	0.3437 (3)	0.5623 (3)	0.29822 (11)	0.0539 (6)	
H19A	0.3275	0.4824	0.2826	0.065*	
H19B	0.2685	0.6179	0.2796	0.065*	
C20	0.2957 (4)	0.5583 (3)	0.35748 (11)	0.0563 (7)	
H20A	0.1746	0.5435	0.3608	0.068*	
H20B	0.3551	0.4919	0.3749	0.068*	
C21	0.3398 (3)	0.6757 (2)	0.38580 (10)	0.0458 (6)	
H21A	0.2847	0.7404	0.3651	0.055*	
C22	0.2693 (4)	0.6842 (2)	0.44366 (10)	0.0553 (7)	
H22A	0.1454	0.6785	0.4406	0.066*	
C23	0.3069 (5)	0.8059 (3)	0.46971 (13)	0.0773 (10)	
H23A	0.2579	0.8082	0.5052	0.116*	
H23B	0.2590	0.8695	0.4480	0.116*	
H23C	0.4277	0.8170	0.4723	0.116*	
C24	0.3247 (5)	0.5812 (3)	0.48076 (12)	0.0782 (9)	
H24A	0.4457	0.5842	0.4857	0.117*	
H24B	0.2938	0.5050	0.4648	0.117*	
H24C	0.2695	0.5894	0.5152	0.117*	

### Atomic displacement parameters (Å^2^)

**Table:**

	*U*^11^	*U*^22^	*U*^33^	*U*^12^	*U*^13^	*U*^23^
O1	0.0850 (15)	0.0717 (11)	0.0416 (9)	−0.0148 (11)	0.0137 (10)	0.0019 (9)
O2	0.0659 (12)	0.0420 (8)	0.0504 (9)	−0.0102 (8)	0.0087 (9)	−0.0097 (8)
N1	0.0661 (14)	0.0536 (11)	0.0370 (10)	−0.0205 (11)	0.0084 (10)	−0.0062 (9)
C1	0.0629 (17)	0.0646 (15)	0.0457 (13)	−0.0236 (14)	0.0031 (13)	−0.0030 (13)
C2	0.078 (2)	0.0529 (13)	0.0566 (16)	−0.0142 (14)	0.0135 (16)	−0.0022 (13)
C3	0.0667 (17)	0.0687 (16)	0.0403 (13)	−0.0044 (14)	0.0025 (13)	−0.0082 (12)
C4	0.0550 (15)	0.0503 (13)	0.0448 (13)	−0.0032 (11)	0.0071 (12)	−0.0088 (11)
C5	0.0461 (13)	0.0393 (10)	0.0357 (11)	−0.0004 (10)	−0.0029 (10)	0.0020 (10)
C6	0.0499 (14)	0.0411 (11)	0.0490 (13)	0.0025 (10)	0.0043 (12)	−0.0013 (11)
C7	0.0593 (16)	0.0358 (11)	0.0525 (14)	−0.0042 (11)	0.0030 (13)	−0.0037 (10)
C8	0.0419 (12)	0.0346 (10)	0.0348 (11)	−0.0007 (9)	−0.0026 (9)	0.0002 (9)
C9	0.0538 (14)	0.0511 (13)	0.0352 (11)	−0.0002 (12)	−0.0066 (11)	−0.0010 (11)
C10	0.0480 (15)	0.0608 (14)	0.0440 (12)	−0.0039 (12)	−0.0159 (11)	−0.0033 (12)
C11	0.0412 (13)	0.0555 (13)	0.0466 (13)	−0.0047 (11)	−0.0083 (11)	−0.0049 (11)
C12	0.0360 (12)	0.0359 (10)	0.0400 (11)	−0.0019 (9)	−0.0018 (10)	−0.0025 (9)
C13	0.0349 (11)	0.0329 (9)	0.0355 (11)	0.0024 (9)	−0.0037 (8)	0.0009 (9)
C14	0.0330 (11)	0.0451 (11)	0.0392 (12)	0.0017 (10)	−0.0043 (9)	0.0036 (10)
C15	0.0401 (13)	0.0548 (13)	0.0396 (12)	−0.0050 (10)	−0.0048 (10)	−0.0014 (11)
C16	0.0381 (12)	0.0364 (10)	0.0385 (11)	0.0008 (9)	−0.0006 (9)	0.0001 (9)
C17	0.0358 (12)	0.0379 (10)	0.0406 (11)	−0.0020 (9)	−0.0004 (10)	0.0000 (10)
C18	0.0514 (14)	0.0454 (11)	0.0401 (12)	0.0020 (10)	0.0006 (11)	−0.0022 (11)
C19	0.0433 (14)	0.0643 (14)	0.0541 (15)	−0.0108 (12)	0.0053 (12)	−0.0092 (13)
C20	0.0472 (15)	0.0633 (15)	0.0585 (15)	−0.0120 (13)	0.0121 (13)	−0.0016 (13)
C21	0.0469 (13)	0.0437 (12)	0.0469 (13)	0.0016 (10)	0.0056 (11)	0.0054 (11)
C22	0.0543 (16)	0.0597 (15)	0.0520 (14)	0.0023 (12)	0.0118 (13)	0.0056 (12)
C23	0.100 (3)	0.0742 (19)	0.0581 (17)	0.0018 (18)	0.0266 (19)	−0.0087 (15)
C24	0.095 (3)	0.085 (2)	0.0553 (17)	0.0004 (19)	0.0128 (17)	0.0227 (16)

### Geometric parameters (Å, °)

**Table:**

O1—C3	1.415 (3)	C11—H11B	0.9700
O1—C2	1.418 (3)	C12—C17	1.539 (3)
O2—C5	1.226 (3)	C12—C13	1.561 (3)
N1—C5	1.378 (3)	C13—C14	1.534 (3)
N1—C1	1.458 (3)	C13—H13A	0.9800
N1—C4	1.466 (3)	C14—C15	1.514 (3)
C1—C2	1.503 (4)	C14—H14A	0.9700
C1—H1A	0.9700	C14—H14B	0.9700
C1—H1B	0.9700	C15—C16	1.517 (3)
C2—H2A	0.9700	C15—H15A	0.9700
C2—H2B	0.9700	C15—H15B	0.9700
C3—C4	1.505 (3)	C16—C17	1.337 (3)
C3—H3A	0.9700	C16—C18	1.510 (3)
C3—H3B	0.9700	C17—C19	1.509 (3)
C4—H4A	0.9700	C18—C21	1.528 (4)
C4—H4B	0.9700	C18—H18A	0.9700
C5—C8	1.551 (3)	C18—H18B	0.9700
C6—C8	1.555 (3)	C19—C20	1.514 (4)
C6—H6A	0.9600	C19—H19A	0.9700
C6—H6B	0.9600	C19—H19B	0.9700
C6—H6C	0.9600	C20—C21	1.513 (4)
C7—C12	1.552 (3)	C20—H20A	0.9700
C7—H7A	0.9600	C20—H20B	0.9700
C7—H7B	0.9600	C21—C22	1.537 (3)
C7—H7C	0.9600	C21—H21A	0.9800
C8—C9	1.562 (3)	C22—C23	1.519 (4)
C8—C13	1.562 (3)	C22—C24	1.524 (4)
C9—C10	1.520 (3)	C22—H22A	0.9800
C9—H9A	0.9700	C23—H23A	0.9600
C9—H9B	0.9700	C23—H23B	0.9600
C10—C11	1.523 (3)	C23—H23C	0.9600
C10—H10A	0.9700	C24—H24A	0.9600
C10—H10B	0.9700	C24—H24B	0.9600
C11—C12	1.545 (3)	C24—H24C	0.9600
C11—H11A	0.9700		
			
C3—O1—C2	109.73 (19)	C17—C12—C13	108.54 (17)
C5—N1—C1	119.21 (19)	C11—C12—C13	107.85 (17)
C5—N1—C4	129.6 (2)	C7—C12—C13	115.32 (19)
C1—N1—C4	110.79 (19)	C14—C13—C12	109.26 (17)
N1—C1—C2	110.7 (2)	C14—C13—C8	115.23 (17)
N1—C1—H1A	109.5	C12—C13—C8	116.57 (17)
C2—C1—H1A	109.5	C14—C13—H13A	104.8
N1—C1—H1B	109.5	C12—C13—H13A	104.8
C2—C1—H1B	109.5	C8—C13—H13A	104.8
H1A—C1—H1B	108.1	C15—C14—C13	109.08 (18)
O1—C2—C1	111.6 (2)	C15—C14—H14A	109.9
O1—C2—H2A	109.3	C13—C14—H14A	109.9
C1—C2—H2A	109.3	C15—C14—H14B	109.9
O1—C2—H2B	109.3	C13—C14—H14B	109.9
C1—C2—H2B	109.3	H14A—C14—H14B	108.3
H2A—C2—H2B	108.0	C14—C15—C16	113.57 (19)
O1—C3—C4	112.3 (2)	C14—C15—H15A	108.9
O1—C3—H3A	109.1	C16—C15—H15A	108.9
C4—C3—H3A	109.1	C14—C15—H15B	108.9
O1—C3—H3B	109.1	C16—C15—H15B	108.9
C4—C3—H3B	109.1	H15A—C15—H15B	107.7
H3A—C3—H3B	107.9	C17—C16—C18	123.2 (2)
N1—C4—C3	109.75 (19)	C17—C16—C15	122.8 (2)
N1—C4—H4A	109.7	C18—C16—C15	114.00 (19)
C3—C4—H4A	109.7	C16—C17—C19	120.5 (2)
N1—C4—H4B	109.7	C16—C17—C12	123.1 (2)
C3—C4—H4B	109.7	C19—C17—C12	116.39 (19)
H4A—C4—H4B	108.2	C16—C18—C21	115.6 (2)
O2—C5—N1	118.7 (2)	C16—C18—H18A	108.4
O2—C5—C8	121.0 (2)	C21—C18—H18A	108.4
N1—C5—C8	120.22 (19)	C16—C18—H18B	108.4
C8—C6—H6A	109.5	C21—C18—H18B	108.4
C8—C6—H6B	109.5	H18A—C18—H18B	107.4
H6A—C6—H6B	109.5	C17—C19—C20	112.8 (2)
C8—C6—H6C	109.5	C17—C19—H19A	109.0
H6A—C6—H6C	109.5	C20—C19—H19A	109.0
H6B—C6—H6C	109.5	C17—C19—H19B	109.0
C12—C7—H7A	109.5	C20—C19—H19B	109.0
C12—C7—H7B	109.5	H19A—C19—H19B	107.8
H7A—C7—H7B	109.5	C21—C20—C19	111.5 (2)
C12—C7—H7C	109.5	C21—C20—H20A	109.3
H7A—C7—H7C	109.5	C19—C20—H20A	109.3
H7B—C7—H7C	109.5	C21—C20—H20B	109.3
C5—C8—C6	110.49 (18)	C19—C20—H20B	109.3
C5—C8—C9	105.57 (17)	H20A—C20—H20B	108.0
C6—C8—C9	114.40 (18)	C20—C21—C18	108.9 (2)
C5—C8—C13	107.80 (17)	C20—C21—C22	113.6 (2)
C6—C8—C13	111.38 (17)	C18—C21—C22	114.7 (2)
C9—C8—C13	106.83 (18)	C20—C21—H21A	106.3
C10—C9—C8	113.76 (19)	C18—C21—H21A	106.3
C10—C9—H9A	108.8	C22—C21—H21A	106.3
C8—C9—H9A	108.8	C23—C22—C24	110.4 (2)
C10—C9—H9B	108.8	C23—C22—C21	112.2 (2)
C8—C9—H9B	108.8	C24—C22—C21	114.3 (2)
H9A—C9—H9B	107.7	C23—C22—H22A	106.4
C11—C10—C9	111.89 (19)	C24—C22—H22A	106.4
C11—C10—H10A	109.2	C21—C22—H22A	106.4
C9—C10—H10A	109.2	C22—C23—H23A	109.5
C11—C10—H10B	109.2	C22—C23—H23B	109.5
C9—C10—H10B	109.2	H23A—C23—H23B	109.5
H10A—C10—H10B	107.9	C22—C23—H23C	109.5
C10—C11—C12	111.9 (2)	H23A—C23—H23C	109.5
C10—C11—H11A	109.2	H23B—C23—H23C	109.5
C12—C11—H11A	109.2	C22—C24—H24A	109.5
C10—C11—H11B	109.2	C22—C24—H24B	109.5
C12—C11—H11B	109.2	H24A—C24—H24B	109.5
H11A—C11—H11B	107.9	C22—C24—H24C	109.5
C17—C12—C11	109.88 (19)	H24A—C24—H24C	109.5
C17—C12—C7	106.60 (18)	H24B—C24—H24C	109.5
C11—C12—C7	108.60 (19)		
